# Rumination in Man

**Published:** 1898-09

**Authors:** N. O’D. Parks

**Affiliations:** Ashton, R.I.


### Rumination in Man.

Ashton, R. I., April 14, 1898.

To the Editor:—I was much interested in Dr. Sinkler's paper on "Merycism or Rumination in Man," published in your issue of the 9th inst., as I recollect that when a young man, say from 18 to 25 years of age, I frequently, at long intervals, used to return my food, especially after a hearty meal when accompanied or more often *followed* by the use of wine or other alcoholic beverage, and finding it rather agreeable, remasticated and restored it to the stomach.

I agree with Dr. Sinkler that rumination is not the result of indigestion but of improper mastication, at least in cases where, as in my own, the food returned is sweet and pleasant, and he is undoubtedly correct in his opinion that it passes out of the stomach and ceases to be returned to the mouth as soon as it becomes fit for intestinal absorption.

N. O'D. Parks, M.D.

